# Nicolau Gregori Czeczko. Former President of the Brazilian College of Digestive Surgery (2017–2018)

**DOI:** 10.1590/0102-672020260000020e1949

**Published:** 2026-08-21

**Authors:** Jurandir Marcondes RIBAS, Osvaldo MALAFAIA

**Affiliations:** 1 Faculdade de Medicina Evangélica Mackenzie do Paraná – Curitiba (PR), Brazil.

 Born on November 4, 1954, in Ponta Grossa, Paraná, Nicolau Gregori Czeczko built a career intertwined with the recent history of digestive surgery in Brazil. The only child of Ukrainian immigrants, he inherited the quiet discipline and steady resilience of his origins. His vocation was awakened in childhood by the Christian values of his maternal grandparents and the constant encouragement given by his father, Alexandre Czeczko.His academic journey began in 1972, when he enrolled at Faculdade Evangélica de Medicina do Paraná (FEMPAR). From the outset, he showed an almost innate inclination for surgery: the operating room became an extension of his training, and his clinical rotations at Hospital Evangélico de Curitiba and Hospital Cajuru de Curitiba were the setting in which his surgical technique and professional character were shaped. He graduated in medicine in 1978 and completed residency training in general and digestive surgery. From that point on, his career gained depth and purpose, establishing him as a leader at Hospital Evangélico de Curitiba. There, he took part in pivotal moments in the modernization of surgical practice, including the institution’s first videolaparoscopic procedure in 1991. His prominence extended beyond the hospital walls, earning him the prestigious state honor known as “*Bicho do Paraná*”. 

**Figure 1:**
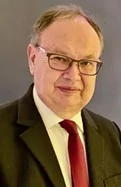


 In 1979, he married Sarah Elizabeth Camargo Augustin, his lifelong partner, with whom he built a strong family: three children (Nicolau Gregori, Alexandre Eduardo, and Letícia Elizabeth) and six grandchildren (Bianca, Alice, Melissa, Eduardo, Augusto, and Lara). Family has not only been a source of support but the very foundation of his journey, the silent axis of meaning and continuity. 

 Dr. Nicolau’s scientific authority extended beyond geographic and intellectual boundaries. His Master’s thesis^4^ ranked first among more than one hundred scientific works presented at the 1st Brazilian Congress of Digestive Surgery, receiving an award from Prof. Dr. Henrique Valter Pinotti (founder of the Brazilian College of Digestive Surgery (CBCD)). In 1992, he completed his Doctoral thesis at Universidade Federal do Paraná (UFPR), in Curitiba, based on a multicenter study conducted in cooperation between Brazil and Germany^5^. The following year, he presented his research in Würzburg, Germany, as an international guest at the Second European Workshop on Compression Anastomosis by Biofragmentable Rings. 

 In 2005, he was elected a Full Academician of the Paraná Academy of Medicine, where he took seat No. 29 with dignity and dedication, becoming a guardian of the profession’s memory and ethical standards. 

 Beyond medicine, Dr. Nicolau found in photography a way of contemplating time. As he himself describes it: “It is the poetic capture of moments in our life that silently reveal the passage of our time.” His sensitive eye as a photographer earned him 31 national and 3 international awards, an aesthetic reflection of the same precision and sensitivity that continue to guide his medical practice to this day. 

 His contribution to the medical literature is extensive and substantial, comprising hundreds of clinical and experimental research articles published in national and international journals^1-3,6,7^, as well as books and book chapters that have become references in the field. This body of work led him to national leadership positions, including the presidency of the CBCD. In that role, he demonstrated not only managerial skills but also a remarkable ability to unite stakeholders, co-chairing the Brazilian Weeks of the Digestive System in 2017 and 2018 alongside leaders of the Brazilian Federation of Gastroenterology (FBG) and the Brazilian Society of Digestive Endoscopy (SOBED). The 2018 edition, held jointly with the XXXIV Pan-American Week of Digestive Diseases, achieved historic attendance, drawing more than 6,850 participants. During his tenure as CBCD President, he actively promoted digestive surgery nationwide. 

 In academic life, he devoted himself intensely to training generations of medical students, surgical residents, and postgraduates, viewing teaching as a natural extension of the medical vocation. He ranked first in the UFPR faculty selection examination in 1986 and began teaching anatomy, having become a Full Professor in 2016 and retiring in 2018. At FEMPAR, also in Curitiba, he held academic leadership roles as Vice-Coordinator and then Coordinator of the Medical Program for 11 years, attaining the rank of Full Professor there in 2018. Since 1994, he has been a faculty member of the Graduate Program in Principles of Surgery. He also taught at Pontifícia Universidade Católica do Paraná (PUCPR) in Curitiba. At the federal level, he served as an evaluator for the Coordination for the Improvement of Higher Education Personnel (CAPES). In all of these roles, he imparted not only knowledge but a lasting formative legacy. 

 His scientific output is robust — not only in volume (208 national and international scientific articles, 3 textbooks, 33 book chapters, dozens of essays, and hundreds of presentations) but also in the continuity and coherence of a vision devoted to advancing surgery. He served on 99 thesis and examination committees and supervised more than one hundred academic works. One of his greatest sources of pride is having personally taught his own children, Alexandre and Letícia, a rare fusion of professional legacy and family heritage. 

 Over decades of clinical, academic, and institutional work, Dr. Nicolau has never strayed from the essence of the medical art: direct, ethical, attentive, and compassionate patient care. His career reaffirms a simple yet profound truth: surgical technique, when divorced from humanity, is incomplete. Conversely, dedication to teaching and to patients, guided by purpose, transcends time. 

 In honoring this trajectory, the ABCD journal celebrates not only the surgeon or the administrator, but above all, the professor, whose presence endures in the generations he trained and the medicine he helped build. 

 As Dr. Nicolau wrote in a publication of the Paraná Academy of Medicine: “Respect is earned, and the pride of service is strengthened through the practice of the art of medicine. It is in that act of dedication — before a patient who cries out for help and places their trust in their physician — that medicine’s mission is renewed and made eternal.” 

## Data Availability

The datasets generated and/or analyzed during the current study are available from the corresponding author upon reasonable request.
